# Tunnel sign on magnetic resonance imaging in neuromelioidosis: A systematic literature review

**DOI:** 10.1016/j.nmni.2025.101639

**Published:** 2025-09-11

**Authors:** Nitin Gupta, Sonali Singh, Tirlangi Praveen Kumar, Sundeep Malla, Astha Sethi, Carl Boodman, Steven Van Den Broucke, Erika Vlieghe, Emmanuel Bottieau, Martin Peter Grobusch, Chiranjay Mukhopadhyay

**Affiliations:** a Department of Infectious Diseases, Kasturba Medical College, Manipal, Manipal Academy of Higher Education, Manipal, 576104, India; b Department of Clinical Sciences, Institute of Tropical Medicine, Antwerp, Belgium; c University of Antwerp, Antwerp, Belgium; d Division of Neurology, Department of Paediatrics, Hospital for Sick Children, University of Toronto, ON, M5G 1X8, Canada; e Department of Radiodiagnosis, Amrita Institute of Medical Sciences, Faridabad, India; f Center of Tropical Medicine and Travel Medicine, Department of Infectious Diseases, Amsterdam University Medical Centers, University of Amsterdam, Amsterdam Public Health – Global Health, Amsterdam Infection & Immunity, Amsterdam, the Netherlands; g Masanga Medical Research Unit (MMRU), Masanga, Sierra Leone; h Centre de Recherches Médicales en Lambaréné (CERMEL), Gabon; i Institut für Tropenmedizin und Deutsches Zentrum für Infektiologie (DZIF), Universität Tübingen, Tübingen, Germany; j Institute of Molecular Medicine and Infectious Diseases, University of Cape Town, Cape Town, South Africa; k Department of Microbiology, Kasturba Medical College, Manipal, Manipal Academy of Higher Education, Manipal, 576104, India

**Keywords:** Melioidosis, CNS melioidosis, *Burkholderia pseudomallei*, Encephalomyelitis, Meningoencephalitis, Microabscesses, Tunnel sign, Magnetic resonance imaging (MRI)

## Abstract

**Background:**

Neuromelioidosis can present with abscesses, meningitis, or encephalomyelitis, but can be missed on blood culture. Linear enhancement of the corticospinal tract (white matter motor pathway) on magnetic resonance imaging (MRI) in the form of a ‘tunnel sign’ is an essential clue for early diagnosis of neuromelioidosis. This systematic review (SR) explores the clinical profile and outcomes of neuromelioidosis patients with tunnel signs.

**Methods:**

An SR was conducted to look for articles reporting individual details of neuromelioidosis patients with tunnel signs (reported or present on published images) on MRI. This review followed PRISMA guidelines and was prospectively registered with PROSPERO (CRD42024597199). After title-abstract and full-text screening, clinical profile and outcome data were extracted and analysed.

**Results:**

Thirty cases (22 articles) with tunnel signs on MRI were included after screening 2985 articles. The traditional risk factors (diabetes mellitus, alcohol intake, steroids, etc.) for melioidosis were present in only 23 % (5/22) of patients. Limb weakness (89 %, 24/27) and cranial nerve involvement (46 %, 11/24) were commonly seen at presentation. Blood and cerebrospinal fluid (CSF) cultures for *B.pseudomallei* were only positive in 15 % (2/13) and 22 % (4/18). Due to low rates of clinical suspicion of neuromelioidosis (25 %, 6/24), empirical steroids and inappropriate antimicrobials were given in 47 % (8/17) and 65 % (9/17) of patients, respectively. A total of 30 % (n = 9) of the patients died.

**Conclusion:**

In melioidosis-endemic areas with access to MRI, recognising the link between the presence of a tunnel sign and neuromelioidosis is crucial to initiate early adequate therapy.

## 1 Background

Melioidosis, caused by the gram-negative bacterium *Burkholderia pseudomallei*, is a systemic infection endemic to tropical regions, particularly Southeast Asia, South Asia, and Northern Australia [1]. While it predominantly affects the lungs, skin, and visceral organs (liver, spleen, prostate), neurological involvement (brain abscesses, extradural/subdural abscesses, meningitis, or encephalomyelitis) is not uncommon and is associated with significant morbidity and mortality [1]. In large cohort studies from Thailand and Northern Australia, 1–2 % of the patients with melioidosis had neurological involvement [2]. Considering the non-specific clinical presentation of neuromelioidosis and relatively lower rates of concurrent bacteremia, it is likely that neuromelioidosis cases are under-reported [3]. A distinct feature of neuromelioidosis on magnetic resonance imaging (MRI) that can help in the diagnosis is the presence of the tunnel sign (longitudinal enhancement of the corticospinal tract), which can help to differentiate it from other causes of encephalomyelitis (post-infectious, autoimmune, paraneoplastic, viral, etc). [4]. An earlier systematic review on neuromelioidosis with 35 cases of encephalomyelitis reported only two cases with linear enhancement without mentioning the relation to the corticospinal tract [5]. This review, therefore, explores the epidemiologic traits, clinical characteristics, treatment modalities, and clinical outcomes of melioidosis cases with corticospinal tract involvement in MRI.

## 2 Methodology

### 2.1 Registration

The review followed the Preferred Reporting Items for Systematic Reviews and Meta-Analyses (PRISMA) guidelines for systematic literature reviews and was registered in the International Prospective Register of Systematic Reviews (PROSPERO CRD42024597199).

### 2.2 Research question

In patients with tunnel signs on MRI in neuromelioidosis, what are the clinical profiles, radiological features, and outcomes?

### 2.3 Search strategy

A systematic bibliographic search was conducted on three databases (Medline/PubMed, Embase, and Web of Science). The following search string was used: (tunnel OR corticospinal OR pyramidal OR neurologic∗ OR brain OR CNS OR nervous OR frontal OR parietal OR occipital OR cerebellar OR white OR cranial OR MRI OR Magnetic) AND (melioid∗ OR Burkholderia OR pseudomallei). All articles with available online full texts published in peer-reviewed journals until 01.06.2024 were included with no restrictions for gender, age group, region, or the language in which it was published. Citation searching was performed to look for additional articles.

### 2.4 Screening and inclusion of articles

Two researchers (NG and SS) separately screened titles and abstracts based on the inclusion/exclusion criteria. Full texts of the articles were independently selected according to the eligibility criteria. All articles describing clinical data relating to human cases of neuromelioidosis on MRI were screened separately for the presence of tunnel signs by a neurologist (SS), a radiologist (SM), and an infectious disease physician (NG or TPK). Discrepancies in the decision to include the study and data extraction were resolved through consensus and by including a third reviewer (CB).

### 2.5 Eligibility criteria

Patients of all ages diagnosed with melioidosis by the reporting clinical team (microbiological, clinic-radiological, or serological) were screened for eligibility. We included articles where individual case details described longitudinal enhancement along the corticospinal tract (tunnel sign) on MRI, or the enhancement was visible on the published image, even if the authors did not report it. All article types with individual case details were included (except for conference abstracts). Those cases where an MRI was not done were excluded.

### 2.6 Data extraction

Two reviewers (NG and SS) extracted data independently in consultation with the team radiologist (SM) using a standardised form containing study characteristics (author, year of publication) and patient demographics. For each variable, only cases where the detail was explicitly reported as present or absent were included in the denominator, with the number reported as present serving as the numerator. Data on epidemiology (geographic area of presentation, diabetes mellitus, other comorbidities), clinical signs and symptoms (fever, hypotension, neurological findings), MRI findings (corticospinal tract involvement, cranial nerve involvement, microabscesses, site of involvement), diagnosis (method of diagnosis and specimen type), and clinical outcomes (death, cure with or without residual neurological deficits) were collected. Cerebrospinal fluid (CSF) cell counts above 5/mm^2^ were considered increased. CSF protein levels of 60 mg/dl or more were considered elevated. Low CSF glucose concentration was defined as lower than two-thirds of the serum glucose concentration. If the corresponding serum glucose concentrations were not mentioned, a glucose concentration of less than 50 mg/dl was taken as low. The data was recorded in a Microsoft Excel spreadsheet.

### 2.7 Critical appraisal

The methodological quality of the included studies was assessed using the Joanna Briggs Institute (JBI) essential appraisal tools for case reports [6].

### 2.8 Data analysis

Qualitative variables were expressed as frequency and percentage. Continuous variables were described as a range. Wherever possible, means (with standard deviation) or medians (with interquartile range) were calculated for quantitative variables.

## 3 Results

### 3.1 Inclusion of articles

**Fig. 1:**
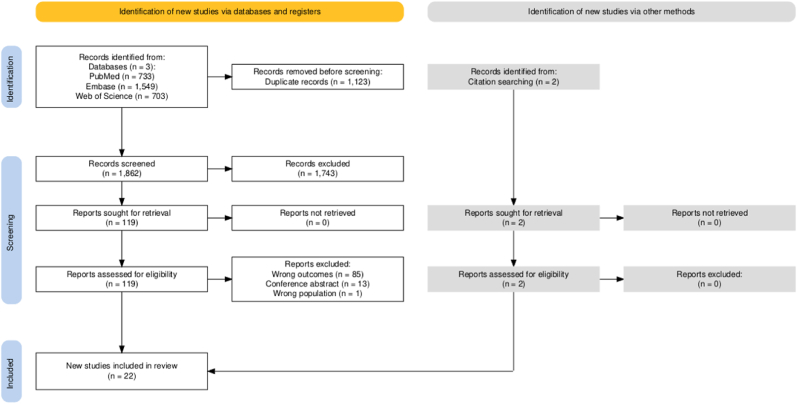
Screening and inclusion of studies with the tunnel sign on magnetic resonance imaging in patients with neuromelioidosis.

A total of 2985 articles were retrieved from PubMed (n = 733), Embase (n = 1549), and Web of Science (n = 703) (Fig. 1). After deleting 1123 duplicate articles, the titles and abstracts of 1862 articles were screened, after which 119 articles were included for full-text screening. Of the 119 articles, 99 were excluded [Wrong outcome (n = 85), conference abstract (n = 13), wrong population (n = 1)]. Two articles were additionally identified from citation searching. A total of 30 cases (22 articles) were included (Supplementary Tables 1–6) [[7], [8], [9], [10], [11], [12], [13], [14], [15], [16], [17], [18], [19], [20], [21], [22], [23], [24], [25], [26], [27], [28]].

### 3.2 Demography

Except for one case from 1992 and one from 2008, all cases were published after 2012, likely because of low access to MRI before that (Supplementary Table 1) [23,28]. Most cases were reported from India (n = 19, 63.3 %) and Australia (n = 7, 23.3 %). Except for eight paediatric cases, all reported cases were more than 18 years of age. Most reported cases were male (n = 22, 73 %) (Table 1). Of the 12 cases where occupation was reported, outdoor occupation was noted in only 25 % (n = 3) (Table 1). Of the 22 cases where host risk factors of acquisition were reported, traditional risk factors such as diabetes (13.6 %, 3/22), steroids (4.5 %, 1/22), and alcohol intake (9.1 %, 2/22) were present in only five (22.7 %) patients (Supplementary Table 1).

**Table 1:** Demography, clinical features, radiological features, diagnosis, and outcomes from articles included in the final analysis.

Subheadings	Variables	Frequency (Percentage)
**Demography and Risk Factors**	Paediatric age group (less than 18 years)	8/30 (27 %)
	Male gender	22/30 (73 %)
	Outdoor occupation	3/12 (25 %)
	Traditional risk factors (diabetes mellitus, alcohol intake, steroids)	5/22 (23 %)
**Clinical presentation**	Presentation within one week of neurological symptom onset	11/24 (46 %)
	Fever	19/25 (76 %)
	Hypotension	0
	Altered sensorium	11/29 (38 %)
	Meningeal signs	9/25 (36 %)
	Limb weakness	24/27 (89 %)
	Cranial nerve involvement	11/24 (46 %)
**MRI findings**	Microabscesses	25/30 (83 %)
	Supratentorial involvement	28/30 (93 %)
	Brain stem involvement	25/30 (83 %)
	Cerebellum involvement	11/30 (37 %)
	Spinal cord involvement	10/30 (33 %)
	Tunnel sign reported by the authors	5/30 (17 %)
**CSF findings**	CSF pleocytosis	17/20 (85 %)
	Lymphocytic predominance	11/16 (69 %)
	Increased protein	16/22 (73 %)
	Decreased glucose concentration	5/22 (23 %)
**Provisional diagnosis**	Tuberculosis	4/17 (23.5 %)
	Autoimmune diseases	5/17 (29.4 %)
	Infectious meningoencephalitis	6/17 (35.3 %)
	Neuromelioidosis	6/24 (25 %)
**Culture positivity**	Blood culture	2/13 (15 %)
	CSF culture	4/18 (22 %)
	Brain biopsy culture	12/12 (100 %)
**Treatment**	Empirical steroids	8/17 (47 %)
	Empirical anti-tuberculous treatment	4/17 (23.5 %)
	Empiric-appropriate antimicrobials∗	6/17 (35 %)
**Outcome (n=30)**	Cure with no ongoing neurologic deficits	11/30 (37 %)
	Death	9/30 (30 %)
	Partial improvement (residual deficits)	10/30 (33 %)

Abbreviations: CSF- cerebrospinal fluid.

∗ Empiric antimicrobial regimen containing ceftazidime or carbapenems (imipenem or meropenem).

### 3.3 Clinical presentation

Of the 24 patients in whom the duration of illness at presentation was reported, 46 % (11/24) presented within a week of symptom onset (Table 1). A total of 76 % (19/25) of patients presented with fever, but none presented with hypotension, a common feature of acute melioidosis (Table 1). Altered sensorium was present in 37.9 % (11/29) of patients, and meningeal signs were present in 36 % (9/25). Of the 27 cases where focal neurological deficits were reported, 88.9 % (24/27) of patients had some limb weakness, which mimicked stroke or acute flaccid paralysis (Table 1). Cranial nerve involvement was seen in 11/24 (45.8 %) patients (Supplementary Table 2).

### 3.4 MRI findings

White matter hyperintensities in T2-weighted images were noted in all patients. The most common locations were supratentorial (93.3 %, 28/30), infratentorial [brain stem (83.3 %, 25/30) cerebellar (36.7 %, 11/30)], or within the spinal cord (33.3 %, 10/30). Apart from the linear corticospinal tract enhancement, microabscesses along the corticospinal tract could be visualised in 83.3 % (25/30) of patients (Table 1). Of the 30 cases where we found a tunnel sign, only 16.6 % (n = 5) of reports mentioned this specific finding (Supplementary Table 3). At least one cranial nerve was involved in 20 % (6/30) (Table 1). The trigeminal nerve was the most commonly involved (16.6 %, 5/30).

### 3.5 CSF findings

CSF findings (partial or complete) were available for 22 patients. CSF cell counts were increased in 85 % (17/20) of the patients, with most cases noting a lymphocyte predominance (68.75 %, 11/16) (Table 1). Of the 17 patients whose CSF cell counts were mentioned, it was less than 100 in 53 % (9/17) (Supplementary Table 4). CSF protein was increased in 72.7 % (16/22) of patients, but the CSF sugar levels were low in only 22.7 % (5/22).

### 3.6 Diagnosis

Neuromelioidosis was suspected before the final diagnosis was established in only 25 % (6/24) of patients (Table 1). TB, autoimmune diseases, and bacterial meningoencephalitis (other than melioidosis) were suspected in 23.5 % (4/17), 29.4 % (5/17), and 35.3 % (6/17) patients, respectively (Table 1). Blood and CSF cultures were positive in only 15.4 % (2/13) and 22.2 % (4/18) of patients. Of the 12 patients for whom a brain biopsy was done, all were culture-positive (Supplementary Table 5). Six patients were diagnosed by culture of respiratory specimens, and one by the culture of tissues obtained from skin and bones. Three patients were diagnosed based on typical clinical and radiological features along with response to treatment, while two were diagnosed with serology (Supplementary Table 5).

### 3.7 Treatment and outcomes

Empirical steroids and anti-tuberculous treatment (ATT) were started in 47 % (8/17) and 4/17 (23.5 %) patients, respectively. Appropriate antimicrobials (regimens containing ceftazidime or carbapenems) were started empirically in only 35.3 % (6/17) of patients (Table 1). A total of 36.7 % (n = 11) patients were cured without residual neurologic deficits, and 30 % (n = 9) patients died. The rest (33.3 %, n = 10) had partial improvement of neurologic symptoms with residual neurological deficits on follow-up. Of the eight patients who received steroids, only 37.5 % (3/8) were cured (Supplementary Table 6). Of the six patients who received appropriate empirical antibiotics, 83.3 % (5/6) were cured. The one person who received appropriate antimicrobial but died had also received empirical steroids (Supplementary Table 6).

### 3.8 Critical appraisal of the included cases

All patients' demographic, diagnostic, and follow-up details were adequately available (Supplementary Table 7). The patients’ past medical history and current clinical presentation condition were absent for 20 % (6/30) and 17 % (5/30) of the patients, respectively. The treatment details were incomplete in 27 % (8/30) of the patients. The details of adverse events were considered unnecessary for the current SR.

## 4 Discussion

### 4.1 Summary of the included studies

This systematic review found 30 cases of neuromelioidosis (including eight paediatric cases) with tunnel signs on MRI published up to 01.06.2024. Traditional risk factors for melioidosis, such as DM, hazardous alcohol use, etc., were absent in 77 % of the patients. Enhancing microabscesses (83 %) along the corticospinal tract were commonly reported in both supratentorial and infratentorial locations. Neuromelioidosis was suspected before culture in only a quarter of the patients. Blood and CSF cultures were frequently negative, and a brain biopsy was often required to confirm the diagnosis. Mortality was reported in 30 %, and a complete cure without ongoing neurologic deficits was described in only 37 % of the patients.

### 4.2 Host and pathogen risk factors for neuromelioidosis

DM and the use of steroids are important risk factors for melioidosis, likely due to impaired neutrophil function, which is vital for the clearance of bacteria. In our review, however, 77 % of the patients had no reported risk factors. In a cohort study from North Australia, DM was less common in neuromelioidosis (8 %) when compared to melioidosis at all sites (36 %) [29]. In the SR by Wongwandee et al., risk factors were absent in 40 % of patients with neuromelioidosis [5]. Previous studies have shown that certain pathogen-related factors can also lead to a predominantly neurological presentation. In a study from Australia, the *bimA*_*Bm*_ allele of the autotransporter protein *bimA* (Burkholderia intracellular motility A) is associated with neurological presentation, brain stem involvement, and encephalomyelitis [30]. None of the included cases reported the presence or absence of the *bimA*_*Bm*_ allele.

### 4.3 Clinical presentation

Owing to the involvement of the corticospinal tract, commonly in the internal capsule, the primary presentation was limb weakness with or without cranial palsies. Many patients had monoparesis or hemiparesis at presentation, which progressed to involve other limbs. This was probably due to the spread of the disease through the commissural fibres [10]. Hsu et al. noted that despite the spread to the contralateral side, the initial infection was more severe, leading to an asymmetry in the weakness [10]. Altered sensorium and meningeal signs were present in approximately one-third of patients each. Cranial nerves, commonly the facial and trigeminal, were involved in nearly half of the patients. Some patients also had corresponding neuralgia or paraesthesia along the distribution of cranial nerves [10,15]. Compared to the traditional descriptions of pyogenic meningitis, where CSF cell counts can frequently be in thousands, CSF counts were not very high in the reviewed cases. The CSF cell counts commonly stayed below 100/mm^2^, rarely exceeded 600/mm^2^, and were frequently lymphocytic. The CSF glucose levels were also often found to be normal. Some authors have drawn parallels between neuromelioidosis and tuberculosis, intracellular bacteria that directly invade the central nervous system and cause lymphocytic pleocytosis with raised protein [31].

### 4.4 Diagnosis of melioidosis

Before the culture result, neuromelioidosis was suspected in only 25 % of the cases. Blood and CSF cultures were frequently negative. The low positivity could result from the sample's paucibacillary nature or an alternative route of spread to the brain (as discussed below). In our SR, only 15 % (2/13) of the patients with a tunnel sign had concurrent bacteremia. It must be noted here that, of the 30 cases, the blood culture report was mentioned in only 13 patients. The relative absence of bacteraemia was also reflected in the absence of shock in any of the included cases, unlike the 21 % shock reported in the Darwin cohort study from Northern Australia [3].

### 4.5 Tunnel sign on MRI

Radiology can serve as a helpful tool for making an early diagnosis of neuromelioidosis. Computed tomography (CT) scans frequently miss neuromelioidosis, especially in acute cases; therefore, MRI is recommended [22,23]. It must be noted that MRI may not be available in several low-resource endemic areas. In a study, CT showed positive findings in only 27 % of patients, whereas MRI was abnormal in all patients [31]. Even in those with positive CT scans, it is tough to differentiate neuromelioidosis from other pathologies [31]. In the report by Jabeen et al., the initial CT led to the misdiagnosis of an infarct, which was later confirmed to be an abscess on MRI [16]. Even though we included cases where an MRI was done, we could not find any cases where a tunnel sign was noted on a CT scan.

**Fig. 2:**
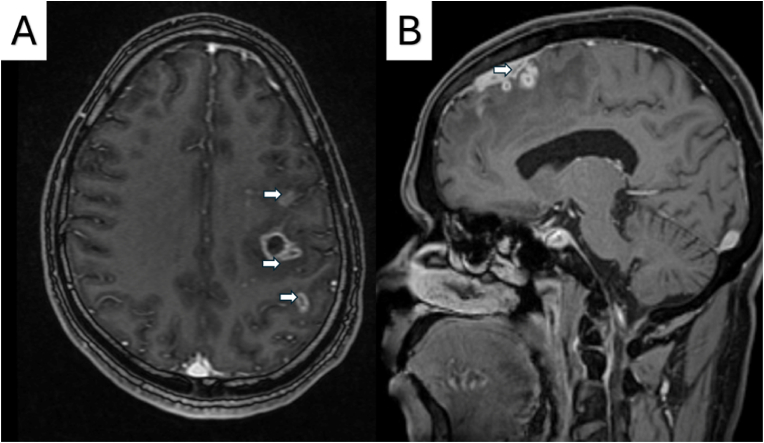
Axial and Sagittal sections of magnetic resonance imaging in patients with neuromelioidosis showing the tunnel sign (white arrow).

A representative panel of tunnel signs on MRI images of two patients (from the archives of the author) with neuromelioidosis is shown in Fig. 2. The white matter hyperintensities on T2-weighted sequences noted in all patients can be easily misdiagnosed as demyelination (post-infection, paraneoplastic, auto-immune, etc.) if the corresponding contrast images (including the thin sections) are not carefully observed. The longitudinal enhancement of the corticospinal tract (tunnel sign) can be noted in the contrast images, and the corresponding thin sections show microabscesses [4]. Of the 17 % that did not report microabscesses in our SR, it was likely missed in some cases because thin sections of MRI were not performed. Also, the microabscesses could be missed if the MRI is performed too early. In the case reported by Ekka et al., the first MRI showed white matter hyperintensities suggestive of acute demyelinating encephalomyelitis (ADEM). Still, the microabscesses could only be appreciated after a few days in the second and third MRIs [14].

**Fig. 3:**
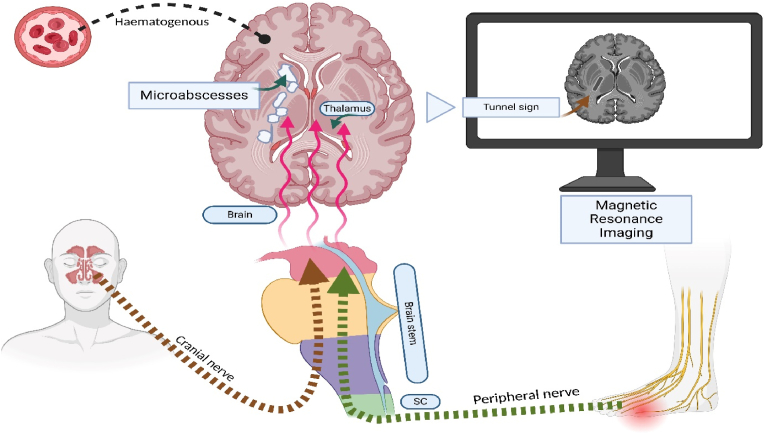
Illustration showing the spread of the bacilli to the brain and appearance of the tunnel sign on imaging The panel's top right side shows the bacilli's haematogenous spread to the brain. The bottom right side of the panel shows bacilli spread through the cranial nerves after inhalation, and the left-sided panel shows bacilli spread through the peripheral nerves and spinal cord after percutaneous inoculation. Bacilli pass through the brain stem and thalamus to form microabscesses along the corticospinal tract, leading to the appearance of a ‘tunnel sign’ on Magnetic Resonance Imaging.(Abbreviation: SC- spinal cord). Image created using Biorender.

Microabscesses in the form of ring-enhancing lesions can be commonly seen in tuberculosis, toxoplasmosis, metastasis, neurocysticercosis, septic emboli, and primary CNS lymphoma [10,15]. Some authors characterised the microabscesses further with a size of 10 mm or less and central restricted diffusion with peripheral enhancement (similar to other pyogenic abscesses) [10,20]. Malignancy commonly has facilitated diffusion in the necrotic centre [20]. Unlike tuberculomas, which display a hypointense or hyperintense centre on T2-weighted imaging, melioidosis frequently has a hyperintense centre [20]. Other differential diagnoses have not described microabscesses along the white matter tracts, as noted in melioidosis [10]. While the presence of a tunnel sign is specific, it is difficult to comment on whether it is sensitive. It should be noted that tunnel signs have also been described in listeriosis and sparganosis, and their local prevalence should be considered when diagnosing neuromelioidosis [15]. The history of ingesting raw meats (frog, snake, fish, etc.), peripheral eosinophilia, or punctate calcification on a CT scan can help diagnose sparganosis [21]. As opposed to the expected low choline peaks on MR spectroscopy in melioidosis, it can be elevated in sparganosis [21].

### 4.6 Routes of neurological involvement in melioidosis (Fig. 3)

Previously, authors have suggested that neurological involvement in melioidosis could be related to exotoxins [28]. This possibility was used to explain the frequent negativity in CSF culture [22,28]. However, subsequent studies showed that neurological involvement results from direct invasion. The bacilli enter the blood through an inhalational or percutaneous inoculation route and cross the blood-brain barrier into the grey-white matter junction [32]. This is supported by reports of brain abscesses in the frontoparietal region that receives the supply from the major middle cerebral artery [26]. The direct invasion can also occur through inoculation in the scalp, followed by skull bone osteomyelitis and intracranial extension [20]. After entering the brain through these routes, the bacilli can traverse down through the corticospinal tracts, forming microabscesses [26]. A third proposed route involves entry through the sinus's olfactory or trigeminal nerve's free nerve endings, followed by intra-axonal transport via cranial or peripheral nerves, with spread through the brainstem and thalamus to the corticospinal tract [20,33]. The frequent sinusitis, hyperintensities noted along the trigeminal nerve, and the frequent involvement of the brain stem and thalamus give some credence to this hypothesis [10]. Bacilli may also spread via the spinal cord, as shown in cases following epidural injection or penetrating limb injury [8]. Initial localized involvement progressed to the brainstem, thalamus, and corticospinal tract, consistent with retrograde transport [8]. Similar peripheral nerve spread has been reported by others [17,24,27].

### 4.7 Treatment and outcome

In the SR, half of the patients received steroids empirically, and only one-third of the patients received appropriate antimicrobials before the diagnosis. Steroids may exacerbate melioidosis, as noted in this previously published report, where reactivation of melioidosis was seen after a patient was given high-dose steroids for COVID-19 [34]. On the other hand, in the absence of confirmation of melioidosis, it is also challenging to avoid steroids in a patient where other differential diagnoses, such as ADEM, TB, or pneumococcal meningitis, are possible, where steroids may be beneficial [35]. Identifying the tunnel sign on MRI early can help avoid an unnecessary and potentially harmful steroid prescription.

The overall outcomes were poor, as only one-third of the patients were cured without ongoing neurologic deficits during the last follow-up. It should be noted that the outcomes are often influenced by other factors not studied in this SR, such as healthcare trajectory before hospitalisation, underlying co-morbidity, other organs affected, level of care received, and the preventive measures employed for secondary healthcare-associated infections.

### 4.8 Limitations of the study

This SR had several limitations, the most important being the small sample size. Data for some variables were missing in several patients; therefore, denominators varied across analyses. As these were case reports that did not consistently follow reporting guidelines, important clinical or treatment details were often incomplete. Relevant (or any) sections of the MRI were not displayed in some cases; therefore, all findings could not be systematically captured. The authors used varied definitions of outcome, which made it difficult to pool them together. Some of the included patients were diagnosed based on clinical, radiological, or serological findings alone, which might not be sufficient to make a definite diagnosis of melioidosis.

## 5 Conclusions

In melioidosis-endemic areas, clinicians should consider neuromelioidosis among patients presenting with cranial nerve palsies, limb weakness, and CSF pleocytosis, even in the absence of traditional melioidosis risk factors. There is a need to improve access and availability of MRI in endemic areas and create awareness about the importance of detecting tunnel signs on MRI.

## CRediT authorship contribution statement

**Nitin Gupta:** Writing – review & editing, Writing – original draft, Visualization, Supervision, Software, Resources, Project administration, Methodology, Investigation, Formal analysis, Data curation, Conceptualization. **Sonali Singh:** Writing – review & editing, Writing – original draft, Methodology, Data curation, Conceptualization. **Tirlangi Praveen Kumar:** Writing – review & editing, Writing – original draft, Data curation, Conceptualization. **Sundeep Malla:** Writing – review & editing, Writing – original draft, Methodology, Data curation. **Astha Sethi:** Writing – review & editing, Methodology. **Carl Boodman:** Writing – review & editing, Methodology. **Steven Van Den Broucke:** Writing – review & editing, Writing – original draft. **Erika Vlieghe:** Writing – review & editing, Writing – original draft. **Emmanuel Bottieau:** Writing – review & editing, Writing – original draft. **Martin Peter Grobusch:** Writing – review & editing, Writing – original draft. **Chiranjay Mukhopadhyay:** Writing – review & editing, Supervision.

## Ethics approval and consent to participate

Not required as this is a systematic review.

## Consent for publication

Not required as this is a systematic review.

## Availability of data and materials

Available from the corresponding author on reasonable request.

## Funding

None.

## Declaration of competing interest

The authors declare that they have no known competing financial interests or personal relationships that could have appeared to influence the work reported in this paper.
