# Supplementary material for: Tunnel sign on magnetic resonance imaging in neuromelioidosis: A systematic literature review
Source: New Microbes New Infect. 2025 Sep 11;68:101639. doi: 10.1016/j.nmni.2025.101639 (PMC12859452; doi:10.1016/j.nmni.2025.101639)
Supplement: Multimedia component 1 [file mmc1.docx]

**Supplementary Files**

Supplementary Table 1: Publication details and demography of included cases with tunnel sign on Magnetic Resonance Imaging

| **Sn** | **Author** | **Year** | **Country** | **Age (years)** | **Gender** | **Occupation** | **Risk factors** |
| --- | --- | --- | --- | --- | --- | --- | --- |
| 1 | Agarwal [7] | 2023 | India | 30 | Male | NA | No |
| 2 | Arif [8] | 2015 | UAE (Native of Srilanka) | 33 | Male | Zoo worker | Recent epidural injection |
| 3 | Chatterjee [9] | 2021 | India | 18 | Male | Student | No |
| 4 | Chia-Tsong Hsu 1 [10] | 2015 | Australia | 43 | Male | NA | NA |
| 5 | Chia-Tsong Hsu 2 [10] | 2015 | Australia | 69 | Female | NA | NA |
| 6 | Chia-Tsong Hsu 3 [10] | 2015 | Australia | 56 | Male | NA | NA |
| 7 | Chia-Tsong Hsu 4 [10] | 2015 | Australia | 41 | Male | NA | NA |
| 8 | Cornelius [11] | 2022 | India | 8 | Female | Student | No |
| 9 | Deuble 1 [12] | 2013 | Australia | 56 | Male | NA | No |
| 10 | Deuble 2 [12] | 2013 | Australia | 41 | Male | Outdoor occupation | Alcohol use |
| 11 | Dimitriou [13] | 2022 | Germany | 47 | Male | Indoor work | Recurrent otitis media, ear surgery |
| 12 | Ekka [14] | 2017 | India | 11 | Female | Student | No |
| 13 | Govindappa 1 [15] | 2022 | India | 32 | Male | NA | NA |
| 14 | Govindappa 2 [15] | 2022 | India | 52 | Male | NA | No |
| 15 | Govindappa 3 [15] | 2022 | India | 41 | Male | NA | NA |
| 16 | Jabeen [16] | 2021 | India | 45 | Male | NA | DM |
| 17 | Maramattom [17] | 2021 | India | 57 | Male | NA | NA |
| 18 | Maulik [18] | 2020 | India | 10 | Male | Student | No |
| 19 | Mishra [19] | 2021 | India | 28 | Female | NA | No |
| 20 | Naik [20] | 2023 | India | 27 | Female | NA | DM |
| 21 | Peh [21] | 2018 | Singapore | 55 | Male | Farmer | DM, CLD (Hep C, alcohol) |
| 22 | Prasanna Kumar [22] | 2016 | India | 1 | Female | NA | No |
| 23 | Samson [23] | 2008 | India | 35 | Male | NA | NA |
| 24 | Shanmugam [24] | 2021 | India | 32 | Female | NA | No |
| 25 | Shobhana A [25] | 2022 | India | 27 | Male | Unemployed | Steroids |
| 26 | Vimal Raj 1 [26] | 2022 | India | 8 | Male | Student | No |
| 27 | Vimal Raj 2 [26] | 2022 | India | 11 | Male | Student | No |
| 28 | Vimal Raj 3 [26] | 2022 | India | 15 | Male | Student | No |
| 29 | Vithoosan [27] | 2022 | Sri Lanka | 47 | Male | NA | No |
| 30 | Woods [28] | 1992 | Australia | 24 | Female | NA | NA |

Abbreviation: Sn- Serial number, NA- Not Available, UAE- United Arab Emirates, DM- Diabetes Mellitus, Hep C- Hepatitis C

Supplementary Table 2: Clinical presentation of included cases with tunnel sign on Magnetic Resonance Imaging

| **Sn** | **Author** | **DOI** | **Presentation** | **Cranial nerve** | **Altered sensorium** | **Meningeal signs** |
| --- | --- | --- | --- | --- | --- | --- |
| 1 | Agarwal [7] | 30 | Hemiparesis (UMN) | VII | No | No |
| 2 | Arif [8] | 3 | Ascending Flaccid Quadriparesis with symmetric axon polyneuropathy | III, IV, V, VI, IX-XII | Yes | No |
| 3 | Chatterjee [9] | 4 | Multiple cranial nerve palsies | III, IV, VI, VII, IX-XII | No | Yes |
| 4 | Chia-Tsong Hsu 1 [10] | NA | NA | NA | No | NA |
| 5 | Chia-Tsong Hsu 2 [10] | NA | NA | NA | Yes | NA |
| 6 | Chia-Tsong Hsu 3 [10] | NA | LL monoparesis | NA | Yes | NA |
| 7 | Chia-Tsong Hsu 4 [10] | NA | LL monoparesis | NA | No | NA |
| 8 | Cornelius [11] | 7 | Hemiparesis | VII | No | Yes |
| 9 | Deuble 1 [12] | 7 | Flaccid hemiparesis | No | No | No |
| 10 | Deuble 2 [12] | 7 | Paraparesis | No | No | No |
| 11 | Dimitriou [13] | 2 | Hemiparesis with urinary incontinence | No | Yes | No |
| 12 | Ekka [14] | 20 | Ascending Flaccid Quadriparesis | No | No | Yes |
| 13 | Govindappa 1 [15] | 5 | Hemiparesis | No | No | No |
| 14 | Govindappa 2 [15] | 10 | Trigeminal neuralgia | V | No | No |
| 15 | Govindappa 3 [15] | 20 | Hemiparesis | No | No | No |
| 16 | Jabeen [16] | 5 | UL monoparesis f/b hemiparesis (flaccid) | VII | Yes | No |
| 17 | Maramattom [17] | 2 | Ascending flaccid quadriparesis with urinary retention | No | No | No |
| 18 | Maulik [18] | 14 | Hemiparesis | IX-XII | Yes | Yes |
| 19 | Mishra [19] | 30 | Spastic hemiparesis | No | No | No |
| 20 | Naik [20] | 15 | NA | NA | Yes | Yes |
| 21 | Peh [21] | 7 | Paraparesis (UMN) | No | Yes | No |
| 22 | Prasanna Kumar [22] | 14 | Cranial nv palsy | III, IV, VI | Yes | No |
| 23 | Samson [23] | NA | UL monoparesis | NA | NA | NA |
| 24 | Shanmugam [24] | 7 | Paraparesis (LMN) | No | Yes | Yes |
| 25 | Shobhana A [25] | 21 | Hemiparesis | No | Yes | No |
| 26 | Vimal Raj 1 [26] | 20 | Quadriparesis (UMN) | III, IV, VI, VII, IX-XII | No | Yes |
| 27 | Vimal Raj 2 [26] | 8 | Quadriparesis | III, IV, VI, VII | No | No |
| 28 | Vimal Raj 3 [26] | 60 | Quadriparesis | No | No | Yes |
| 29 | Vithoosan [27] | NA | Hemi f/b quadriparesis (flaccid) | No | No | No |
| 30 | Woods [28] | 70 | Hemiparesis | VII | No | Yes |

Abbreviation: Sn- Serial number, DOI- Duration of illness in days, NA- Not Available, LL- Lower limb, UL- Upper limb, f/b- followed by UMN- upper motor neuron, LMN- lower motor neuron, nv-nerve, lower cranial- lower cranial nerves (9,10,11,12), eye movt- nerves involved in eye movement (3,4,6)

Supplementary Table 3: Magnetic Resonance Imaging features of neuromelioidosis patients with tunnel sign

| **Sn** | **Author** | **CST** | **Microabscesses** | **Supra** | **Brainstem** | **Cerebellum** | **Cranial nv** | **Spinal cord** | **Tunnel mentioned** |
| --- | --- | --- | --- | --- | --- | --- | --- | --- | --- |
| 1 | Agarwal [7] | Yes | Yes | Yes | Yes | No | No | No | Yes |
| 2 | Arif [8] | Yes | Yes | Yes | Yes | No | No | Yes | No |
| 3 | Chatterjee [9] | Yes | Yes | Yes | Yes | No | V, VI, VII | No | No |
| 4 | Chia-Tsong Hsu 1 [10] | Yes | Yes | Yes | Yes | Yes | No | No | No |
| 5 | Chia-Tsong Hsu 2 [10] | Yes | Yes | Yes | No | No | No | No | No |
| 6 | Chia-Tsong Hsu 3 [10] | Yes | Yes | Yes | Yes | Yes | 5 | No | No |
| 7 | Chia-Tsong Hsu 4 [10] | Yes | Yes | No | Yes | Yes | No | Yes | No |
| 8 | Cornelius [11] | Yes | Yes | Yes | Yes | No | No | No | No |
| 9 | Deuble 1 [12] | Yes | Yes | Yes | Yes | No | No | No | No |
| 10 | Deuble 2 [12] | Yes | No | Yes | Yes | No | No | Yes | No |
| 11 | Dimitriou [13] | Yes | Yes | Yes | Yes | Yes | No | No | No |
| 12 | Ekka [14] | Yes | Yes | Yes | No | No | No | No | No |
| 13 | Govindappa 1 [15] | Yes | Yes | Yes | Yes | No | No | No | Yes |
| 14 | Govindappa 2 [15] | Yes | Yes | No | Yes | Yes | V | No | Yes |
| 15 | Govindappa 3 [15] | Yes | Yes | Yes | Yes | Yes | No | No | Yes |
| 16 | Jabeen [16] | Yes | Yes | Yes | No | No | No | No | No |
| 17 | Maramattom [17] | Yes | No | Yes | Yes | No | No | Yes | No |
| 18 | Maulik [18] | Yes | No | Yes | Yes | Yes | No | Yes | No |
| 19 | Mishra [19] | Yes | Yes | Yes | Yes | No | No | No | No |
| 20 | Naik [20] | Yes | Yes | Yes | Yes | No | No | No | No |
| 21 | Peh [21] | Yes | Yes | Yes | No | No | No | No | Yes |
| 22 | Prasanna Kumar [22] | Yes | Yes | Yes | Yes | No | III | No | No |
| 23 | Samson [23] | Yes | Yes | Yes | No | No | No | No | No |
| 24 | Shanmugam [24] | Yes | Yes | Yes | Yes | No | No | Yes | No |
| 25 | Shobhana A [25] | Yes | Yes | Yes | Yes | No | No | Yes | No |
| 26 | Vimal Raj 1 [26] | Yes | Yes | Yes | Yes | Yes | V | Yes | No |
| 27 | Vimal Raj 2 [26] | Yes | Yes | Yes | Yes | Yes | No | Yes | No |
| 28 | Vimal Raj 3 [26] | Yes | Yes | Yes | Yes | Yes | No | No | No |
| 29 | Vithoosan [27] | Yes | No | Yes | Yes | No |  | Yes | No |
| 30 | Woods [28] | Yes | No | Yes | Yes | Yes | No | No | No |

Abbreviation: Sn- Serial number, NA- Not Available, CST- corticospinal tract involved, nv- nerves, Cranial nv- The number of cranial nerves involved in imaging, Tunnel mentioned- tunnel sign mentioned in the manuscript

Supplementary Table 4: Cerebrospinal fluid findings of included patients with tunnel sign

| **Sn** | **Author** | **CSF cells** | **Pleocytosis pattern** | **CSF protein** | **CSF glucose** |
| --- | --- | --- | --- | --- | --- |
| 1 | Agarwal [7] | Increased | lymphocytic | Increased | Low |
| 2 | Arif [8] | Increased | lymphocytic | Increased | Normal |
| 3 | Chatterjee [9] | Increased | neutrophilic | Normal | Normal |
| 4 | Cornelius [11] | Normal | NA | Normal | Normal |
| 5 | Deuble 1 [12] | Increased | lymphocytic | Normal | Normal |
| 6 | Deuble 2 [12] | Increased | lymphocytic | Increased | Low |
| 7 | Dimitriou [13] | Increased | NA | Increased | Low |
| 8 | Ekka [14] | Increased | lymphocytic | Increased | Normal |
| 9 | Govindappa 1 [15] | NA | NA | Normal | Normal |
| 10 | Govindappa 2 [15] | NA | NA | Increased | Normal |
| 11 | Govindappa 3 [15] | Increased | lymphocytic | Increased | Normal |
| 12 | Maramattom [17] | Increased | lymphocytic | Increased | Normal |
| 13 | Maulik [18] | Increased | lymphocytic | Increased | Normal |
| 14 | Mishra [19] | Increased | lymphocytic | Increased | Normal |
| 15 | Prasanna Kumar [22] | Normal | Normal | Normal | Normal |
| 16 | Shanmugam [24] | Increased | neutrophilic | Increased | Low |
| 17 | Shobhana A [25] | Increased | lymphocytic | Increased | Low |
| 18 | Vimal Raj 1 [26] | Increased | lymphocytic | Increased | Normal |
| 19 | Vimal Raj 2 [26] | Normal | NA | Increased | Normal |
| 20 | Vimal Raj 3 [26] | Increased | neutrophilic | Normal | Normal |
| 21 | Vithoosan [27] | Increased | neutrophilic | Increased | Normal |
| 22 | Woods [28] | Increased | neutrophilic | Increased | Normal |

Abbreviation: Sn- Serial number, NA- Not Available, CSF- cerebrospinal fluid

Supplementary Table 5: Modalities used for diagnosis in included cases with the tunnel sign

| **Sn** | **Author** | **NM suspect b4 CS** | **Working diagnosis** | **Diagnosis** |
| --- | --- | --- | --- | --- |
| 1 | Agarwal [7] | NA | NA | Brain Bx Culture |
| 2 | Arif [8] | No | GBS, TB, listeriosis | Respiratory Culture |
| 3 | Chatterjee [9] | No | meningitis | CSF culture |
| 4 | Chia-Tsong Hsu 1 [10] | No | NA | Blood culture |
| 5 | Chia-Tsong Hsu 2 [10] | No | NA | Brain Bx Culture |
| 6 | Chia-Tsong Hsu 3 [10] | No | NA | Respiratory Culture |
| 7 | Chia-Tsong Hsu 4 [10] | No | NA | Respiratory Culture |
| 8 | Cornelius [11] | No | Bells palsy, meningitis | Clinicoradiologically |
| 9 | Deuble 1 [12] | Yes | NA | Respiratory Culture |
| 10 | Deuble 2 [12] | No | NA | Respiratory Culture |
| 11 | Dimitriou [13] | No | Meningitis | CSF and Brain Bx Culture |
| 12 | Ekka [14] | No | ADEM, meningitis | Brain Bx Culture |
| 13 | Govindappa 1 [15] | NA | NA | Brain Bx Culture |
| 14 | Govindappa 2 [15] | Yes | melioidosis | CSF PCR |
| 15 | Govindappa 3 [15] | No | TB | Brain Bx Culture |
| 16 | Jabeen [16] | Yes | melioidosis | Brain Bx Culture |
| 17 | Maramattom [17] | No | Demyelinating disease, neurosarcoidosis | Brain Bx Culture |
| 18 | Maulik [18] | Yes | melioidosis | Serology |
| 19 | Mishra [19] | No | TB, vasculitis | Brain Bx Culture |
| 20 | Naik [20] | NA | NA | Blood culture |
| 21 | Peh [21] | No | Sparganosis | Brain Bx Culture |
| 22 | Prasanna Kumar [22] | No | NA | Skin and bone culture |
| 23 | Samson [23] | NA | NA | Brain Bx Culture |
| 24 | Shanmugam [24] | No | TB | CSF culture |
| 25 | Shobhana A [25] | No | Meningitis | Brain Bx Culture |
| 26 | Vimal Raj 1 [26] | NA | NA | Serology |
| 27 | Vimal Raj 2 [26] | Yes | Melioidosis | Clinicoradiologically |
| 28 | Vimal Raj 3 [26] | Yes | Melioidosis | Clinicoradiologically |
| 29 | Vithoosan [27] | No | Neuromyelitis Optica | CSF culture |
| 30 | Woods [28] | NA | NA | Respiratory Culture |

Abbreviation: Sn- Serial number, NA- Not Available, NM suspect b4 CS- Neuromelioidosis was suspected before the culture results by the author, GBS- Guillain Barre Syndrome, TB- tuberculosis, ADEM- Acute demyelinating encephalomyelitis, CSF- cerebrospinal fluid, PCR- Polymerase chain reaction assay

Supplementary Table 6: Management and outcomes in included cases

| **Sn** | **Author** | **Empirical steroids** | **Appropriate empirical antibiotic** | **Specific treatment** | **Final Outcome** |
| --- | --- | --- | --- | --- | --- |
| 1 | Agarwal [7] | NA | NA | mero, tmp-smx | Cured |
| 2 | Arif [8] | No | No | mero, tmp-smx | Partial improvement |
| 3 | Chatterjee [9] | No | No | mero, tmp-smx | Death |
| 4 | Chia-Tsong Hsu 1 [10] | NA | NA | NA | Partial improvement |
| 5 | Chia-Tsong Hsu 2 [10] | NA | NA | NA | Death |
| 6 | Chia-Tsong Hsu 3 [10] | NA | NA | NA | Death |
| 7 | Chia-Tsong Hsu 4 [10] | NA | NA | NA | Death |
| 8 | Cornelius [11] | Yes | No | mero, tmp-smx, caz | Cured |
| 9 | Deuble 1 [12] | NA | NA | mero | Death |
| 10 | Deuble 2 [12] | NA | NA | mero | Death |
| 11 | Dimitriou [13] | No | No | caz | Partial improvement |
| 12 | Ekka [14] | Yes | Yes | Caz, tmp-smx | Cured |
| 13 | Govindappa 1 [15] | NA | NA | NA | Cured |
| 14 | Govindappa 2 [15] | No | Yes | NA | Cured |
| 15 | Govindappa 3 [15] | Yes | No | mero, tmp-smx | Cured |
| 16 | Jabeen [16] | No | NA | mero, caz | Partial improvement |
| 17 | Maramattom [17] | Yes | Yes | mero | death |
| 18 | Maulik [18] | NA | Yes | mero, doxy | Cured |
| 19 | Mishra [19] | Yes | No | mero,caz, doxy | Partial improvement |
| 20 | Naik [20] | NA | NA | NA | Death |
| 21 | Peh [21] | Yes | No | Caz, mero, tmp-smx | Partial improvement |
| 22 | Prasanna Kumar [22] | No | No | mero, tmp-smx | Cured |
| 23 | Samson [23] | NA | NA | Caz, tmp-smx | Partial improvement |
| 24 | Shanmugam [24] | Yes | No | mero | Death |
| 25 | Shobhana A [25] | No | No | mero, tmp-smx | Cured |
| 26 | Vimal Raj 1 [26] | NA | NA | mero | Partial improvement |
| 27 | Vimal Raj 2 [26] | No | Yes | mero | Cured |
| 28 | Vimal Raj 3 [26] | No | Yes | mero, caz | Cured |
| 29 | Vithoosan [27] | Yes | No | mero,tmp-smx | Partial improvement |
| 30 | Woods [28] | NA | NA | Caz, chloramphenicol | Partial improvement |

Abbreviation: Sn- Serial number, NA- Not Available, mero- meropenem, tmp-smx- trimethoprim-sulphamethoxazole, caz- ceftazidime, doxy-doxycycline

Supplementary Table 7: JBI checklist for critical appraisal of case reports

| **Sn** | **Author** | **Demography** | **History** | **Presentation** | **Diagnosis** | **Treatment** | **Follow-up** | **Adverse event** |
| --- | --- | --- | --- | --- | --- | --- | --- | --- |
| 1 | Agarwal [7] | Yes | Yes | Yes | Yes | Yes | Yes | Not applicable |
| 2 | Arif [8] | Yes | Yes | Yes | Yes | Yes | Yes | Not applicable |
| 3 | Chatterjee [9] | Yes | Yes | Yes | Yes | Yes | Yes | Not applicable |
| 4 | Chia-Tsong Hsu 1 [10] | Yes | No | No | Yes | No | Yes | Not applicable |
| 5 | Chia-Tsong Hsu 2 [10] | Yes | No | No | Yes | No | Yes | Not applicable |
| 6 | Chia-Tsong Hsu 3 [10] | Yes | No | No | Yes | No | Yes | Not applicable |
| 7 | Chia-Tsong Hsu 4 [10] | Yes | No | No | Yes | No | Yes | Not applicable |
| 8 | Cornelius [11] | Yes | Yes | Yes | Yes | Yes | Yes | Not applicable |
| 9 | Deuble 1 [12] | Yes | Yes | Yes | Yes | Yes | Yes | Not applicable |
| 10 | Deuble 2 [12] | Yes | Yes | Yes | Yes | Yes | Yes | Not applicable |
| 11 | Dimitriou [13] | Yes | Yes | Yes | Yes | Yes | Yes | Not applicable |
| 12 | Ekka [14] | Yes | Yes | Yes | Yes | Yes | Yes | Not applicable |
| 13 | Govindappa 1 [15] | Yes | Yes | Yes | Yes | No | Yes | Not applicable |
| 14 | Govindappa 2 [15] | Yes | Yes | Yes | Yes | No | Yes | Not applicable |
| 15 | Govindappa 3 [15] | Yes | Yes | Yes | Yes | Yes | Yes | Not applicable |
| 16 | Jabeen [16] | Yes | Yes | Yes | Yes | Yes | Yes | Not applicable |
| 17 | Maramattom [17] | Yes | Yes | Yes | Yes | Yes | Yes | Not applicable |
| 18 | Maulik [18] | Yes | Yes | Yes | Yes | Yes | Yes | Not applicable |
| 19 | Mishra [19] | Yes | Yes | Yes | Yes | Yes | Yes | Not applicable |
| 20 | Naik [20] | Yes | No | No | Yes | No | Yes | Not applicable |
| 21 | Peh [21] | Yes | Yes | Yes | Yes | Yes | Yes | Not applicable |
| 22 | Prasanna Kumar [22] | Yes | Yes | Yes | Yes | Yes | Yes | Not applicable |
| 23 | Samson [23] | Yes | No | Yes | Yes | No | Yes | Not applicable |
| 24 | Shanmugam [24] | Yes | Yes | Yes | Yes | Yes | Yes | Not applicable |
| 25 | Shobhana A [25] | Yes | Yes | Yes | Yes | Yes | Yes | Not applicable |
| 26 | Vimal Raj 1 [26] | Yes | Yes | Yes | Yes | Yes | Yes | Not applicable |
| 27 | Vimal Raj 2 [26] | Yes | Yes | Yes | Yes | Yes | Yes | Not applicable |
| 28 | Vimal Raj 3 [26] | Yes | Yes | Yes | Yes | Yes | Yes | Not applicable |
| 29 | Vithoosan [27] | Yes | Yes | Yes | Yes | Yes | Yes | Not applicable |
| 30 | Woods [28] | Yes | Yes | Yes | Yes | Yes | Yes | Not applicable |
